# Spontaneous Encoding of Event Roles in Hominids

**DOI:** 10.1162/opmi_a_00202

**Published:** 2025-04-22

**Authors:** Sarah Brocard, Pavel V. Voinov, Balthasar Bickel, Klaus Zuberbühler

**Affiliations:** Department of Comparative Cognition, Institute of Biology, University of Neuchatel, Neuchatel, Switzerland; Center for the Evolutionary Origins of Human Behavior (EHUB), Kyoto University, Kyoto, Japan; Institute for the Interdisciplinary Study of Language Evolution (ISLE), University of Zurich, Zurich, Switzerland; School of Psychology & Neuroscience, University of St Andrews, St Andrews, Scotland (UK)

**Keywords:** comparative cognition, language evolution, event cognition, event roles

## Abstract

When observing social interactions, humans rapidly and spontaneously encode events in terms of agents, patients and causal relations. This propensity can be made visible empirically with the switch cost paradigm, a reaction time experiment and well-established tool of cognitive psychology. We adapted the paradigm for non-human primates to test whether non-linguistic animals encoded event roles in the same way. Both human and non-human participants were requested to attend to different social interactions between two artificially coloured (blue or green) actors and to target the actor masked by a specified colour (e.g., blue), regardless of her role. We found that when we switched the targeted colour mask from agents to patients (or vice versa) the processing time significantly increased in both hominid species (i.e., human and chimpanzee), suggesting that event roles were spontaneously encoded and subsequently interfered with our simplistic colour search task. We concluded that the propensity to encode social events in terms of agents and patients was a common feature of hominid cognition, as demonstrated in several human and one chimpanzee participant, pointing towards an evolutionarily old and phylogenetically shared cognitive mechanism central to language processing.

## INTRODUCTION

Understanding social interactions is fundamental for survival and a well-documented predictor of fitness (e.g., Alberts, 2019; Cheney et al., 2016; Silk, 2007), although relatively little is known about the underlying representational mechanisms. Extensive research with humans has consistently revealed a predisposition to attending and processing social interactions in the visual domain (McMahon & Isik, 2023). This may be grounded in a hardwired neurobiological predisposition to represent facing dyads, as a fundamental feature of social perception (Papeo, 2020). Indeed, the visual system prioritises socially-interacting over non-interacting pairs (Papeo et al., 2019; Skripkauskaite et al., 2023; Su et al., 2016; Vestner et al., 2019, 2020). This ability emerges early since infants as young as 6 months already demonstrate heightened efficiency in detecting facing compared to non-facing dyads (Goupil et al., 2022; Papeo & Abassi, 2019; Papeo et al., 2017). Privileged perception of social interactions appears to be a phylogenetically old predisposition (Deen et al., 2023), with evidence that even newly hatched domestic chicks (*Gallus gallus*) exhibit a preference for (seemingly socially) interacting pairs of lights over non-interacting ones (Zanon et al., 2024).

The perceptually grounded ability to process social interactions is intertwined with a propensity to perceive the world as causally structured. A classic demonstration is Michotte's (1963) launching effect, by which an object *A* collides with a resting object *B*, thereby giving the impression of causality, i.e., that *A* caused *B*'s motion. This ‘*illusion of causality*’ (Michotte, 1963; Scholl & Tremoulet, 2000) emerges early in life (Galazka & Nyström, 2016; Leslie, 1982; Leslie & Keeble, 1987) and has been replicated with various action types (Gao et al., 2009; White & Milne, 1997) highlighting a core feature of human cognition independent of age and culture (Mascalzoni et al., 2013; Morris & Peng, 1994; Rimé et al., 1985). Here again, the propensity to perceive causal interactions as special appears to be older than humans, with various animal studies having reported a sensitivity to causal relations in the mechanical domain (Cacchione & Krist, 2004; Hanus, 2009; Mascalzoni et al., 2010; O’Connell & Dunbar, 2005).

In the social domain, causal events can generally be decomposed in terms of event roles as agent or patient, each occupying a specific function in relation to the action. *The dog chasing the postman* and *the postman chasing the dog* involve the same entities and action but differ in terms of roles, or ‘*who does what to whom*'. It has been argued that event roles are abstract categories that can be universally discriminated and generalised to any entity, as long as it can adopt agent (doer) or patient (receiver) characteristics (Dowty, 1991; Rissman & Majid, 2019), an ability that emerges in early infancy (Hamlin et al., 2007; Robertson & Suci, 1980). Some animals also keep track of event roles, both in the visual (Krupenye & Hare, 2018) and auditory domain (Bergman et al., 2003; Clay et al., 2016; Slocombe et al., 2010; Slocombe & Zuberbühler, 2005; see Wilson et al., 2022 for a review). A recent study further demonstrated that great apes, when tasked with actively selecting between agents or patients of previously watched events, consistently made decisions influenced by the nature of the protagonists (i.e., animate or inanimate), as well as the nature of the interaction (Brocard et al., 2024). Overall, there is very little doubt that animals discriminate agents from patients, further suggesting evolutionary roots that predate the emergence of the human lineage, a finding with particular relevance for evolutionary theories of language (Wilson et al., 2022).

A peculiar feature of human event cognition is the speed and spontaneity by which event roles are identified (Dobel et al., 2007; Vettori et al., 2025). Humans can extract different types of social interactions as rapidly as the blink of an eye (Hafri et al., 2013; Isasi-Isasmendi et al., 2023). In one study, Hafri et al. (2018) asked human participants to react as rapidly as possible to an arbitrary visual feature in a depiction of people interacting with each other (e.g., always select the person wearing a *blue* shirt). Remarkably, if the person with the blue shirt suddenly switched from being the agent to being the patient (or the reverse), response latencies increased significantly, suggesting that the event roles were spontaneously encoded ‘along the way’ and interfered with the task at hand, the so-called ‘switch cost effect’ (Jerslid, 1927). Switch cost effects have been used in a range of cognitive tasks as they are excellent tools to bring to light unconscious or conflicting mental processes (Hafri et al., 2018; Jerslid, 1927; Oosterwijk et al., 2012; Pecher et al., 2003; Rogers & Monsell, 1995; Spence et al., 2001; Stoet & Snyder, 2007; Vettori et al., 2025; Wiseheart et al., 2016).

A few studies with non-human animals have also used switch cost paradigms, but mainly to study low-level visual processing (pigeons: Castro & Wasserman, 2016; Meier et al., 2013, 2016; E. M. O’Donoghue et al., 2020; E. O’Donoghue & Wasserman, 2021; macaques: Avdagic et al., 2014; Caselli & Chelazzi, 2011; Smith & Beran, 2018; Stoet & Snyder, 2007). Here, we were interested in higher-level cognition, that is, whether great apes, if asked to attend to arbitrary low-level visual features, would also encode event roles, even if not specifically asked to do so, as had been demonstrated in humans (Hafri et al., 2018). Demonstrating that non-human primates, similar to humans, can rapidly and automatically extract the structure of social events through visual processing of the world would suggest that event roles are likely to be routinely and readily available to primate observers. This availability could serve as common ground, forming the foundation for encoding events in terms of roles within language. By contrast, if structuring events in terms of agent and patient roles required effortful deliberation, it would be less likely for multiple individuals to generate them simultaneously, potentially hindering the emergence of a shared external language.

## METHODS

### Experimental Design

Humans and many animal species can recognise and correctly interpret still pictures, even without previous experience (see Bovet & Vauclair, 2000 for a review; hamadryas baboons: Kyes & Candland, 1987; long-tailed macaques: Kyes et al., 1992; rhesus monkey: Rosenfeld & Van Hoesen, 1979; chimpanzees: Gardner & Gardner, 1984; Hayes & Hayes, 1953; Savage-Rumbaugh et al., 1980) and if they convey motion information this can even create a sense of causality (adult humans: Freyd, 1983; Guterstam & Graziano, 2020; Kourtzi & Kanwisher, 2000; human infants: Shirai & Imura, 2016; rhesus macaques: McFarland et al., 2013). This literature encouraged us to adapt the original switch-cost protocol by Hafri et al. (2018) for great apes. To this end, we also capitalized on recent findings that hominids view and interpret events in terms of agent and patient roles (Brocard et al., 2024; Wilson et al., 2024), and presented pictures of pairs of chimpanzees, gorillas, humans or orangutans interacting in an agent-patient relationship (e.g., grooming, playing, touching). We then arbitrarily masked the agent or the patient with blue or green shading colours that great apes like and discriminate easily (Pene et al., 2020; Wells et al., 2008). In contrast to Hafri et al. (2018), the stimuli were presented on a touchscreen allowing direct selection of a target, rather than pressing a button.

We first conducted a pilot study (see Supplementary Text S1) with two orangutans, which allowed us to fine-tune the final training and test protocols. Problems recognised during the pilot study were the large number of trials required to obtain a reward as well as the strong effect of the repetition of the target side. We thus decided to present the stimuli in pairs (instead of sequences of several stimuli) to enhance the likelihood of selecting the correct assigned target and to limit the effect of the repetition of the target’s side.

### Subjects

#### Humans.

Ten undergraduate students (*N* = 7 females; mean 21.0 ± 1.3 years old; range [19; 24]) from the University of Neuchâtel (Switzerland) were recruited via e-mail. All participants had normal or corrected-to-normal vision and none of them were colour-blind. Before the start of the experiment, participants received basic information about the procedure and received a detailed information sheet. They were instructed to find a (randomly assigned) coloured target on images depicting two interacting individuals in order to touch it as rapidly and accurately as possible. All participants were naive to the purpose of the study, signed an informed consent form, completed a short questionnaire and attended a debriefing and question and answer session after the experiment. The entire experiment took approximately 2 hours to complete, with short breaks between blocks. Participants were offered CHF 40 for their participation.

#### Great Apes.

Subjects were recruited from a group of *N* = 15 chimpanzees and *N* = 7 gorillas housed at Basel Zoo, Switzerland. All individuals had access to indoor and outdoor enclosures (chimpanzees: 767 m^2^; gorillas: 753 m^2^) that contained ropes, hammocks, climbing structures and freshly provided material to build nests. Individuals were fed a mix of fruit and vegetables supplemented with small amounts of proteins, with several feeds distributed throughout the day and free access to water.

A total of *N* = 6 chimpanzees (*N* = 4 females; mean 13.3 ± 15.1 years old; range [4; 42]) and *N* = 4 gorillas (*N* = 3 females; mean 15.5 ± 12.3 years old; range [7; 33]) participated in the study. However, the training procedure turned out to be extremely arduous and discouraging for great apes (as it was for humans), to the effect that only one chimpanzee, Kume, the 19-years old alpha male, completed the entire study. While this is an obvious shortcoming, Kume’s stamina and eventual successful completion is informative of the cognitive capacities of chimpanzees as a species.

#### Ethical Statement.

This study was non-invasive, risk-free and participation was on a voluntary basis for both species. The study was approved by the Ethics Committee of the University of Neuchâtel (project 101/2022), the Cantonal Veterinary Office of Basel Stadt (permit cantonal number 3077) and the Animal Welfare Officer at Basel Zoo.

### Stimuli

**Figure 1.**
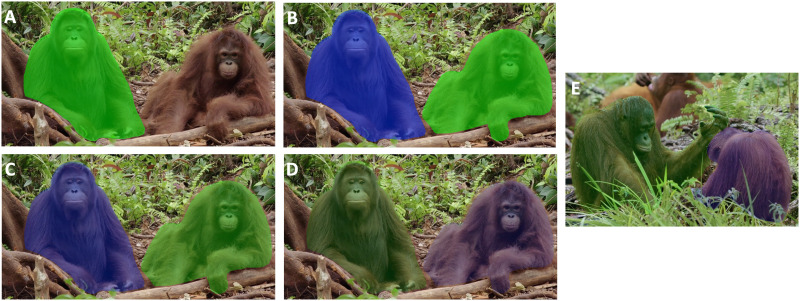
**Example of stimuli (A–D) for the training phase 1 to 4 and (E) for the test**. (A) Training phase 1: only targeted colour with a 50% transparency mask, as many touch as needed to reach the target, feedback in every trial, session of 20 trials. (B) Training phase 2: two actors coloured with 50% transparency mask, only one touch allowed to correctly press the target, feedback on all trials, 30 trials per daily session. (C) Training phase 3: same as phase 2 but transparency of the mask set at 70%. (D) Training phase 4: transparency of the masks set at 85%, daily session of 40 trials and sound reward at every trial but food only delivered every two trials with a new sound. (A-D) A single image is presented in its four possible configurations across the four phases. (E) Test: transparency of the masks set at 85%, and sound reward at every trial but food only delivered every two trials with a new sound. Photo credit: Orangutan Jungle School, Season #1, NHNZ Worldwide.

Stimuli were coloured photographs of two adult individuals from the same species (i.e., chimpanzees, gorillas, humans or orangutans) interacting in eight different ways (test trials; for details of event categories see Table S1) or just being next to each other (training trials). The identities of the actors varied across nearly all stimuli, with limited repetition and in human stimuli only. All participants were exposed to all stimuli, i.e., actors of their own and the three other species. *N* = 40 images were used for the test (10 images per actor species) and *N* = 60 images for the training (15 images per species). Semi-transparent coloured masks were superimposed to both actors, either blue (RGB [0, 0, 255]) or green (RGB [0, 255, 0]), with transparency set to 85% maximum (Figure 1D–E), which resulted in four combinations, i.e., agent left or right and in blue or green.

### Touchscreen Equipment

Data were collected weekdays from 8:00 to 18:00 local time, from September 2022 to February 2024. The ape setup consisted of a touchscreen (Iiyama ProLite T1931SR, 19”, 1280 × 1024 resolution, 5ms response time, resistive technology) connected to a laptop computer (Dell Latitude 7420) allowing to display the stimuli with Matlab’s Psychophysics Toolbox Version 3 (Brainard, 1997; Pelli, 1997). For the chimpanzees, the setup was fixed in one enclosure and protected by a customised Plexiglas box (73 × 55 × 35 cm) open on its bottom allowing the apes to access the screen and retrieve the rewards. For the gorillas, the screen was attached to the mesh of the enclosure at the start of each session and removed at the end (Figure S1). The touchscreen was calibrated every day before the start of the session with eGalaxTouch software (v.5.14.0.19810). Human data were also collected via a laptop (Lenovo Thinkpad T15) connected to a touchscreen (Iiyama ProLite T2553Mis-B1, 24”, 1920x1080 resolution, 4 ms response time, infrared technology).

### Training Phase

A target colour (blue or green) was randomly assigned to each participant at the beginning of the study. Humans were verbally instructed to select the targeted colour, whereas great apes underwent training. This consisted of rewarding the subjects to consistently select targets of any actor species (all stimuli were presented in randomised order) masked by the assigned colour. Training consisted of four phases. In phase 1, only one actor was applied a mask (transparency 50%; Figure 1A) from the targeted colour and participants were rewarded at every correct pressing. To pass this phase 1, participants needed an accuracy of at least 80% in one 20 trials session. In phase 2, the second actor was also coloured (Figure 1B) and participants only had one chance to correctly select the right target, with an 80% accuracy on a 30 trials session required to pass this phase. The difficulty of the remaining two phases increased progressively as the transparency of the mask increased, making it more difficult to detect the target. In phase 3, the transparency increased to 70% (Figure 1C), and by the final phase 4, it was set to 85% (Figure 1D). In addition, in phase 4, food rewards were no longer delivered after each correct trial, but only after two correct trials, which was indicated by a new sound. Training was considered completed when participants obtained an accuracy of 80% in a session of 40 trials in phase 4. The number of trials per session was gradually increased because the great ape subjects were not used to completing so many trials in a single day.

*N* = 6 chimpanzees and *N* = 4 gorillas participated in the training, but as mentioned, only one chimpanzee successfully completed all four training phases (dropouts, phase 1: *N* = 3; phase 2: *N* = 1; phase 3: *N* = 2; phase 4: *N* = 3), mostly due to lack of concentration and motivation. We opted against further prolonging the training, recognising that the crucial aspect for detecting a switch cost lies in attentiveness to the stimuli.

The *N* = 10 human participants were verbally instructed to pay attention to the actor masked by the assigned colour. Human participants could therefore skip the first three phases of the training and only completed *N* = 10 trials of phase 4 (Figure 1D).

### Testing Phase

#### Chimpanzee.

For the chimpanzee we generated a list of *N* = 3,200 trials, sorted into four randomised blocks (based on the actor’s species depicted on the stimuli) of 800 trials each. Importantly, we ensured that the participant saw as many pairs of stimuli with *(i)* the target on the same side with the same role, *(ii)* the target on the same side with a switched role, *(iii)* the target on the switched side with the same role, or *(iv)* the target with switched side and role. We also ensured that no more than two pairs of the same configuration followed one another. Each image was presented 80 times (20 presentations of the same configuration of the stimuli, a configuration being for example the agent in green on the left-hand side and the patient in blue on the other side, Figure 1E). As the identities of the actors varied in nearly all stimuli, no role-switching within event categories were implemented. Based on the results of the pilot study (see Supplementary Text S1), stimuli were presented as consecutive pairs of trials with a food reward after each pair, instead as sequences of multiple trials.

**Figure 2.**
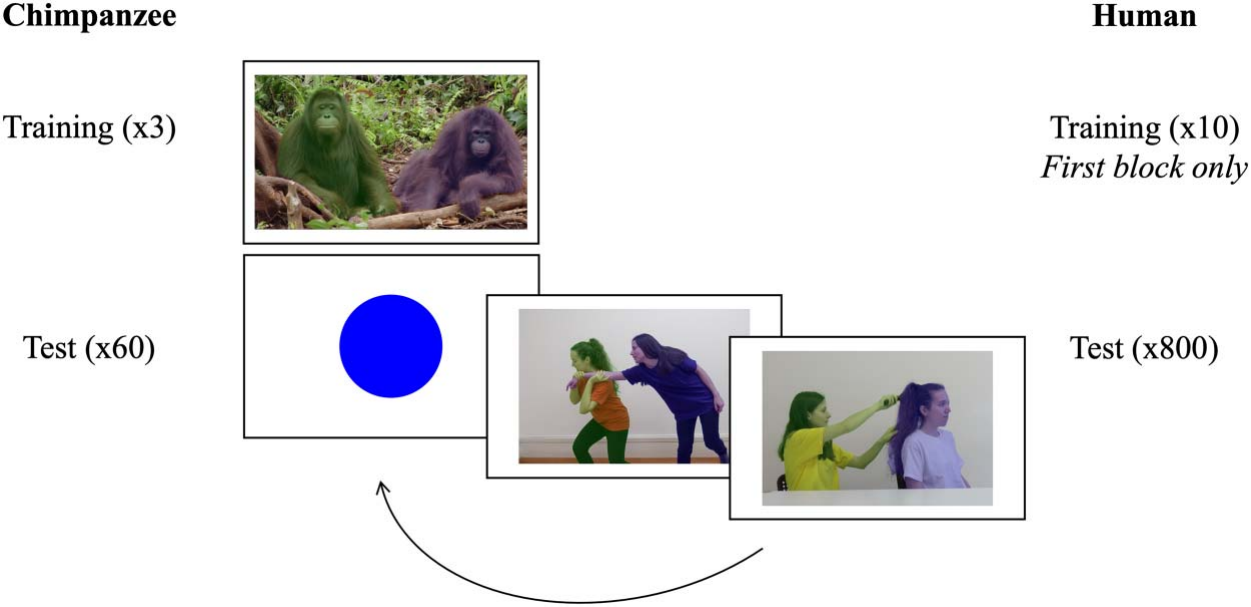
**Schematic illustration of the test session structure.** Before starting the test, the chimpanzee completed three training trials while humans completed 10 training trials, all of phase 4 (Figure 1D). It was followed by 60 test trials for the chimpanzee and an entire block (*N* = 800 trials) for the humans. Images were presented in pairs starting with a circle of the targeted colour. At the end of a pair, a “food sound” was emitted and chimpanzees received a food reward. The images were displayed full screen and the circle at 600 × 600 pixels. Photo credit: (training) Orangutan Jungle School, Season #1, NHNZ Worldwide; (tests) A. Isasi-Isasmendi, S. Sauppe, and C. Andrews.

A test session started with the chimpanzee pressing the uniformly-lit, green touchscreen, which triggered a positive sound and food delivery. The participant then completed three warm-up trials (i.e., images randomly positioned on the screen) and three training trials from phase 4 (Figure 1D). The participant then saw a circle of his targeted colour that he had to press to get access to the test session of *N* = 60 trials. Each trial consisted of an image depicting an agent acting on a patient and the participant was requested to select the actor masked by his targeted colour, which triggered sound feedback (i.e., correct or incorrect) and led to the next trial independently of the success. Once the participant made its second choice, again a sound feedback was produced, followed by a “food sound” accompanied with the delivery of a piece of food. Following this, the circle of the targeted colour reappeared, which the participant needed to press to get access to the next two trials. After completion of the *N* = 60 trials, the screen turned red, and the participant received three pieces of food to indicate the end of the session (Figure 2).

#### Humans.

For human participants, we generated unique lists of *N* = 3,200 trials each, following the exact same procedure as for the chimpanzee. Human participants were tested in the same way as the chimpanzee, although they did not get warm-up trials but transitioned directly from the training phase 4 to the test trials, and they did not receive food reward but money compensation at the end of the experience. In another difference, human participants were not tested in sessions, rather they completed each block consisting of *N* = 800 trials (Figure 2), after which the participant was allowed to have a break, before the next block of *N* = 800 trials and so forth.

Reaction times (RTs) were determined as the time intervals from the stimulus display until the screen was touched and released. Accuracy was defined as the success in selecting the correct assigned colour target.

### Data Processing

After removal of the first trials of a pair (as there was no switch cost possible), trial exclusion criteria were similar to those used by Hafri et al. (2018), namely: trials in which more than one press on the screen was necessary to reach a target (correct or incorrect), trials faster than 200 ms, trials with RTs slower or faster than 2.5 standard deviations of each subject’s mean RT and trials with incorrect answers (kept for the accuracy analyses). This resulted in 54.0% trials excluded for the chimpanzee and an average of 5.7% trials excluded for human participants.

A preliminary analysis showed that human participants found it easier to detect blue than green actors, regardless of their event role (see Supplementary Text S2). Actors coloured in green were generally harder to detect, possibly because the natural surroundings in the photos were often of a greenish colour. We therefore split the dataset in two, and only report the performance of participants assigned the blue targets in the main results, which was consistent with the assigned colour of the single chimpanzee that successfully completed the training.

### Data Analysis

Statistical analyses were conducted in R (R Core Team, 2021, v.4.0.5) using Bayesian generalised linear models with the *brms* (Bürkner, 2017, 2018, 2021) to Stan (Carpenter et al., 2017). This approach was chosen because we aimed to quantify evidence for and against differences between species, whereas frequentist methods only allow for the rejection of a null hypothesis.

Bayesian Bernoulli models with logit links were fitted to model *(i)* accuracy and *(ii)* response side. For the intercepts and all population-level predictors, normally distributed priors were assigned (*μ* = 0, *σ* = 1.5), resulting in relatively flat distributions on the logit scale. Exponentially distributed priors (*λ* = 1) were used for the standard deviation of group-level effects. When modelling the accuracy, we included as predictors of interest the species of the participants, the repetition of the target’s side and role, along with their interaction, the event role of the target and the side of the agent. To control for potential confounding factors, features of the stimuli (i.e., the colour of the agent, species of the actors, and event category; see Table S2 for formal definitions of all variables) were included as fixed effects, as well as random slopes by stimuli, and block. When not modelled as predictor of interest the side of the agent and the role of the target were also added as random slopes. Then, in modelling the response side, our primary focus was on identifying trial features that could influence choices. Therefore, we included the species of the participant and its interaction with block, side of the agent, role of the target, and event category as fixed effects. Additionally, participants and stimuli were treated as random intercepts, with side and colour of the agent as random slopes for the stimuli.

Reaction times were modelled by fitting Bayesian regressions, using exponentially modified Gaussian distributions (Baayen & Milin, 2010), with logarithmic links functions for both sigma and beta. Normally distributed priors (*μ* = 0, *σ* = 2.5) were used for the intercept, all population-level predictors and the residual standard deviation sigma, exponentially distributed priors (*λ* = 1) were used for the standard deviation of group-level effects, and finally gamma distributed priors (*k* = 1, *θ* = 0.2) were used for the regression coefficient beta. The predictors of interest were the species of the participants, repetition of the target’s side and role, as well as their interactions. Additional population-level predictors were included to control for potential confounding effects (i.e., the side of the agent, the role of the target, the colour of the agent, species of the targets, and event category). Finally, these same effects were used as random slopes by stimuli, blocks and participants. Additional models were considered to assess the effect of blocks and for human participants only, to control for the effect of the target colour (see Supplementary Text S2).

For each outcome variable, several models were fitted with different combinations of fixed predictors. To determine the best fitting model for each outcome variable, these models were compared using their expected log pointwise predictive density (elpd) under leave-one-out cross validation, which was approximated by importance sampling from the posterior (Vehtari et al., 2017; Yao et al., 2018). A higher elpd indicates a higher out-of-sample predictive fit, thus a better model. The convergence of all models was ensured through trace plots, R-hat values, and effective sample size (ESS) diagnostics. Posterior predictive checks were additionally used to visually assess the fit between observed data and data simulated by the model.

For all predictors of interest, i.e., the population-level (fixed) effects (*β*), we report their medians and 90% credible intervals. Additionally, we report the posterior probability of the hypothesis that an estimate is either smaller or larger than 0 (or 0.5 depending on the specific hypothesis), denoted as P(*β* < 0) or similar, to quantify the directional evidence for the hypothesis. To illustrate the differences between parameters levels while controlling for all other variables, marginal effects were computed using the emmeans package (Lenth et al., 2018). These marginal effects were expressed either as contrasts on the response scale—indicating the direct difference in predicted outcomes between parameter levels—or as odds ratios, which describe the relative odds of an event occurring under different parameter conditions. Throughout the analyses, an effect was considered robust if at least 90% of posterior estimates were either above chance (0.5) or different from 0, i.e., P(*P* > 0.5) ≥ 0.90 or P(*β* > 0) ≥ 0.90.

## RESULTS

### Accuracy

In the test trials, participants saw photographs with two hominids (chimpanzees, gorillas, humans or orangutan) engaged in a social interaction with one actor coloured in blue and the other in green (Figure 1E; *N* = 40 photographs). By means of vocal instruction or training, participants were instructed to always select the actor tints with their assigned colour, regardless of its event role. As explained above, we only analysed participant responses which were assigned blue targets.

After data processing, we found a main difference in the accuracy between humans and the chimpanzee. While humans were highly accurate in selecting the blue target (mean accuracy*_Humans_* = 99.5% ± 7.1%), the chimpanzee struggled much more with this task (mean accuracy*_Chimpanzee_* = 66.3% ± 47.3%).

Since humans were so highly accurate, we proceeded with analysing the chimpanzee data only and found that the best fitting model (see Table S3 for all comparisons) only included the event role of the target as predictor, and accuracy was better when the target was the patient (marginal effect: median*_(Role Target = Patient) – (Role Target = Agent)_* = 2.206 odds ratio, 90% CI = [1.119; 4.437], meaning that the chimpanzee’s odds of correctly choosing the target when it was a patient were approximately double the odds as when it was an agent; posterior median P*_Role Target = Agent_* = 0.312, 90% CI = [0.188; 0.480], P(*P_Role Target = Agent_* < 0.5) = 0.97; posterior median P*_Role Target = Patient_* = 0.689, 90% CI = [0.523; 0.813], P(*P_Role Target = Patient_* > 0.5) = 0.97).

### Switch Cost Effects - Spatial Continuity and Event Roles

If participants were capable of keeping track of targets from one trial to the next, we predicted an increase in reaction time in responding to the blue target, if it unexpectedly switched sides, in breach of a basic feature of reality: the spatial continuity of bodies. Second, if participants also kept track of the event roles assigned to them by the social interaction, we predicted an increase in reaction time in responding to the blue target, if its event role unexpectedly switched from agent to patient or vice versa.

Humans and the chimpanzee had similar reaction times in selecting their blue targets (median RT*_Humans_* = 747.2 ms; median RT*_Chimpanzee_* = 764.3 ms).

**Figure 3.**
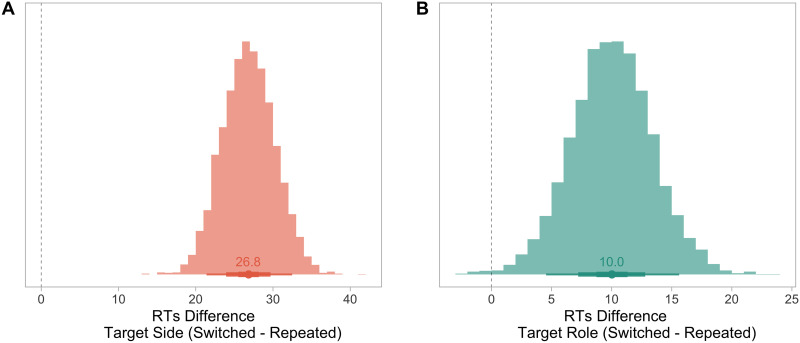
**Posterior probabilities of reaction time differences arising from the target switching (A) the side and (B) the event role.** Plotted data are posterior probabilities from the best fitting model of the RTs differences of the target’s event role and side in the photographs. The dashed line corresponds to no difference (0). The points represent the median of the estimates (values specified above) and the three levels of thickness of the point intervals represent the 30, 60 and 90% credible intervals.

Including repetitions of the target’s side and the target’s role as additive effects, without the species of the participants, slightly improved the fit of the model to the data, as shown by model comparisons (Δelpd*_Best model vs. Model with the Participant’s Species_* = −0.5, SE_Δ*elpd*_ = 0.7; see Table S4). This best fitting model revealed an effect of the repetition of the target’s event role (*β_Repetition Target Role_*: median = −4.99, 90% CI = [−8.30; −1.73], P(*β* < 0) = 1) and an effect of the repetition of the target’s side (*β_Repetition Target Side_*: median = −13.44, 90% CI = [−16.76; −10.16], P(*β* < 0) = 1; Figure 3).

Interfering with the spatial arrangement had a considerably stronger impact on processing time than interfering with the event role of a social interaction (Table 1). In particular, if the target suddenly switched sides from left to right (or vice versa) from one trial to the next, then reaction times of all participants were impacted (medians: Δ*_Humans_* = 54 ms slower; Δ*_Chimpanzee_* = 48 ms faster), with no robust statistical difference between species (marginal effect when the side was repeated: median*_Humans – Chimpanzee_* = −17.9, 90% CI = [−323; 308]; marginal effect when the side was switched: median*_Humans – Chimpanzee_* = 4.01, 90% CI = [−310; 324]). This demonstrates that participants identified targets individually and tracked their spatial position within the social interaction.

**Table 1.** Participants’ median reaction time changes if spatial position or event roles were kept identical or switched from one trial to the next, including switch costs and median marginal effects from the best fitting model

Condition	Participant's species	Reaction times (ms ± SD)		Switch cost (ms)	Median marginal effect (90% CI)
		Repeated	Switched		
Side	Human	723.3 (342)	777.4 (352)	54.1	26.9 [21.3; 32.4]
	Chimpanzee	791.3 (939)	743.4 (846)	−47.9	
Role	Human	744.3 (349)	750.6 (347)	6.32	9.98 [4.68; 15.5]
	Chimpanzee	757.4 (863)	773.9 (934)	16.5	

*Note*. Reaction times were extracted from the raw data, while median marginal effects were extracted from the best model and are the difference between *Switched* and *Repeated*. The switch cost was defined as the difference between reaction times to switched and repeated spatial location or event role, respectively.

Importantly, if the event role of the target suddenly switched from one trial to the next, then reaction times increased for all participants, but again with no robust statistical difference between the two species (medians: Δ*_Human_* = 6 ms; Δ*_Chimpanzee_* = 16 ms; marginal effect when the role was repeated: median*_Humans – Chimpanzee_* = 4.05, 90% CI = [−312; 330]; marginal effect when the role was switched: median*_Humans – Chimpanzee_* = −18.0, 90% CI = [−337; 305]), demonstrating that all participants processed the event roles of the targets in the depicted social interaction (Table 1).

Both species were faster if the target was an agent than a patient (medians: Δ*_Human_* = 17 ms; Δ*_Chimpanzee_* = 49 ms), although this effect was not statistically robust (marginal effect: median*_Agent – Patient_* = −10.5, 90% CI = [−58.4; 37.9]). Finally, the chimpanzee was faster if the target changed from agent to patient relative to a change from patient to agent (median Δ = 21 ms), while humans were slower (median Δ = 10 ms), but not robustly so (marginal effect: median_*(Agent to Patient) – (Patient to Agent)*_ = −10.4, 90% CI = [−57.7; 36.0]).

### Switch Cost Effects - Conspecific Actors Only

When considering the subset of trials with social interactions performed by conspecifics only (humans responding to human interactions, the chimpanzee responding to chimpanzee interactions), model comparison (Table S5) showed that including the participant's species, the repetition of the target's event role, the repetition of the target's side, as well as their interactions, improved the fit of the model to the data (Δelpd*_Best fitting model vs. Null model_* = −10.9, SE_Δ*elpd*_ = 6.8). This best fitting model revealed an interaction between the target’s event role and side (*β_Repetition Target Role × Repetition Target Side_*: median = 53.4, 90% CI = [31.1; 75.5], P(*β* > 0) = 1), an interaction between the species of the participant and the repetition of the target role (*β_Human × Repetition Target Role_*: median = −24.0, 90% CI = [−47.0; −1.4], P(*β* < 0) = 0.96), as well as a triple interaction between the species of the participant, the repetition of the target role and side (*β_Human × Repetition Target Side × Repetition Target Role_*: median = −55.8, 90% CI = [−79.0; −32.7], P(*β* < 0) = 1).

Humans paid a switch cost if both spatial continuity and event role were disrupted between two consecutive trials (marginal effect: median_(*Role switched × Side switched) – (Role repeated × Side repeated)*_ = 39.8, 90% CI = [23.3; 58.2]), while the chimpanzee did not (marginal effect: median_*(Role switched × Side switched) – (Role repeated × Side repeated)*_ = −21.9, 90% CI = [−82.8; 34.9]). However, the chimpanzee experienced a positive effect (faster reaction times) when only the role changed and spatial continuity was maintained (marginal effect: median_*(Role switched × Side repeated) – (Role repeated × Side repeated)*_ = −145.4, 90% CI = [−208.3; −81.8]), while humans experienced a negative effect (slower reaction times) when only the side changed and role of the target was maintained (marginal effect: median_*(Role repeated × Side switched) – (Role repeated × Side repeated)*_ = 35.4, 90% CI = [18.5; 52.4]).

### Other Effects

Similar to the two orangutans in the pilot study (see Supplementary Text S1), the side of the response (i.e., pressing on the left or right hand side) was better explained by the side of the agent than by the null model (Δelpd*_Best fitting model vs. Null model_* = −8.2, SE *_Δelpd_* = 3.8), but robustly only for the chimpanzee (chimpanzee: marginal effect median*_Agent Left – Agent Right_* = 1.515 odds ratio, 90% CI = [1.163; 1.926], meaning that the chimpanzee’s odds of selecting the target on the right side (the dependant variable) was approximately 1.5 times higher if the agent was on the left (opposite) side. In other words, the chimpanzee demonstrated a preference for selecting the patient over the agent; posterior median P*_Chimpanzee select the actor on the right side × Side Agent Right_* = 0.385, 90% CI = [0.326; 0.447], P(*P*_*Chimpanzee select the actor on the right side × Side Agent Right*_ < 0.5) = 1; humans: marginal effect median*_Agent Left – Agent Right_* = 0.954 odd ratio, 90% CI = [0.899; 1.016], posterior median P*_Human select the actor on the right side × Side Agent Right_* = 0.614, 90% CI = [0.552; 0.673], P(*P*_*Human select the actor on the right side × Side Agent Right*_ > 0.5) = 1). This result for the chimpanzee was also accompanied by a side bias (ratio left/right = 1.62), which might explain the low accuracy rate and the high proportion of data removal.

The blocks also had an effect on the reaction times, with statistically robust interaction with the repetition or switch of the target’s role and side (Δelpd*_Model with interactions vs. Model without interactions_* = −5.0, SE_Δ*elpd*_ = 4.7). Participants had statistically robust longer reaction times in the first block only (marginal effects: median*_Block1 − Block2_* = 75.0, 90% CI = [14.6; 139.9]; median*_Block1 − Block3_* = 112.0, 90% CI = [15.7; 203.3]; median*_Block1 − Block4_* = 129.2, 90% CI = [67.5; 190.9]; median*_Block2 − Block3_* = 39.8, 90% CI = [−45.3; 120.8]; median*_Block2 − Block4_* = 57.0, 90% CI = [−16.7; 125.1]; median*_Block3 − Block4_* = 16.1, 90% CI = [−66.1 ; 98.9]; see Table S6 for all contrasts with the interaction).

## DISCUSSION

The present study aimed to investigate whether great apes, similar to humans, rapidly and spontaneously encoded event roles when required to find a coloured target in images depicting natural social interactions of an agent acting on a patient. Even though only one non-human primate, a chimpanzee, completed the full study, including training and tests, our study generated very similar results for the chimpanzee and the humans. Both species were slower to select targets when their roles changed, relative to when their roles remained across consecutive stimuli, suggesting that all participants spontaneously encoded the event structure by processing the event roles (i.e., agent, patient) played by the actors, aligning with findings from human psychology (Hafri et al., 2018).

This switch cost effect appeared to be stronger in non-human primates, despite no robust statistical differences, with the chimpanzee taking approximately 4 ms (median marginal effect) longer than humans to respond when the role of the target switched. The human reaction times were however consistent with previously reported results (Hafri et al., 2018; Vettori et al., 2025). Regardless of the magnitude of the cost, the data suggest that both species processed event roles rapidly and spontaneously and that such processing induced a cognitive cost when the role of the target switched. More generally, the finding supports the hypothesis that the ability to decompose events in terms of event roles might not be uniquely human but shared with other primates, indicating deep evolutionary roots of event cognition (Wilson et al., 2022). Our study adds to this theory, suggesting that the ability to automatically assign event roles might have emerged as a spontaneous cognitive routine in early hominids, and possibly also other groups of animals. As such, it may have played a key role as a blueprint for the rapid event encoding mechanism that underlies grammatically structured language.

In both species participants also experienced a switch cost when the spatial continuity of actors was disrupted between consecutive trials (i.e., if *A* was to the left of *B* and then to the right of *B* on the next trial). Although interfering with the spatial arrangement appeared to incur a substantial switch cost, even more so for humans if the conspecifics’ roles also changed, requiring participants to recode information on both dimensions. Social interactions are not just facing dyads, but also grounded in a place (Papeo, 2020; Papeo et al., 2019; Skripkauskaite et al., 2023; Su et al., 2016; Vestner et al., 2019, 2020), suggesting that detection and understanding of social events also require locational information. It is reasonable to assume that before processing event roles, the brain first encodes the spatial disposition, which might explain why both species in the current study demonstrated a larger switch cost for spatial re-arrangement than event roles disruption. A recent study further demonstrated that altering visual properties (i.e., body postures) from one stimulus to the next induced a switch cost effect in humans (Vettori et al., 2025).

Finally, some human participants may have had difficulties discriminating between agents and patients in the ape interactions, which might have resulted in some switch cost differences between the chimpanzee and the humans. Such difficulties in agent/patient discrimination might have prevented the emergence of a switch cost, resulting in an overall lower switch cost. This might also explain why human participants were slower to answer when the side of the target switched from one stimulus to the next, while the chimpanzee and human participants in similar studies were faster (Hafri et al., 2018; Vettori et al., 2025).

Despite similarities in reaction times, the accuracy data revealed notable differences between the humans and the chimpanzee. Humans demonstrated near-perfect accuracy, whereas the chimpanzee showed significantly lower accuracy. This difference may be attributed to several factors, including differences in training between species, task familiarity, attentional capacities, interest in the task, and lack of understanding of the task. Despite these concerns, the chimpanzee participant passed all training thresholds, went through warm-up training trials prior to the tests, and received sound feedback throughout the sessions. A more plausible explanation of the difference might be related to the lack of attention or the rush to complete a sequence to receive the food reward. In addition, in a task switching paradigm, Stoet and Snyder (2007) stressed that human participants receiving training (ca. a third of that for monkeys in their study) had an increased switching cost effect for reaction times but a decreased switching cost effect for the accuracy. Thus, the large number of training trials (ca. 1000) underwent by the chimpanzee participant might have prevented the emergence of switch cost effect in accuracy and might explain the seemingly stronger – compared to human participants in this study – switch cost effect on reaction times. The chimpanzee's lower accuracy might also be influenced by his side bias, which could have impacted overall performance.

Interestingly, the chimpanzee was more accurate when the target had a patient role compared to an agent role, suggesting two non-exclusive possible interpretations. One possibility is that the chimpanzee wrongly learned to associate the target with the patient role rather than with the colour blue. However, this seems highly unlikely, as the training stimuli were specifically designed to exclude any agent-patient relationships, making it near impossible for the chimpanzee to have formed such an association. An alternative explanation is that patients might be inherently easier to process, reducing cognitive demands and minimizing interference with the target detection task. This interpretation could also explain why the chimpanzee was faster to respond when the target’s role switched from agent to patient but was slower when the target switched from patient to agent.

One limitation of this study, already pointed out, is that our results are based on a single chimpanzee. This limits the generalizability of the findings, especially when previous studies have stressed individual variations in sensitivity to switching cost effect (Caselli & Chelazzi, 2011; Stoet & Snyder, 2007). However, conducting such a protocol with non-human animals - voluntarily participating and under no water or calorie restrictions - remains a tedious task as participants need to remain focused and motivated for long periods of time. Such a difficulty appeared quite clearly with the lower accuracy rate of the chimpanzee compared to humans, and even more so with the number of participants that dropped the training. Recently, Papeo et al. (2024) used habituation and pupillometry methods to test 7-month-olds infants’ reaction to switching event roles. Such methods could prove highly advantageous for adapting similar protocols to non-human great apes, as they require minimal training and significantly fewer trials.

Additionally, due to the challenges encountered during data collection, this study lacks a control condition, such as a “back-to-back” scenario. Previous research (Hafri et al., 2018; Vettori et al., 2025) has suggested that changes in posture and orientation may account for at least some of the observed switch-cost effects, though not all. Hence, it would be valuable in future research to explore whether, like humans, non-human great apes exhibit similar sensitivities to body posture and orientation when processing and assigning event roles.

In conclusion, this study provides preliminary evidence that a non-human primate, like humans, rapidly and spontaneously encoded event roles from social interactions, as indicated by the role switch cost effect. This finding potentially suggests that the cognitive ability to automatically process agents and patients in social events is evolutionarily ancient and shared with our closest living relatives. The paradigm has demonstrated how hard it is to override or even impossible to ignore a deeply anchored propensity to process events in terms of agents causing changes in patients, in both human and a non-human hominid, providing valuable insights into the early evolution of the language faculty eventually capable of syntax (Zuberbühler, 2019, 2022; Zuberbühler & Bickel, 2022).

## Acknowledgments

We thank Andrea Cruz Alegria for helping in data collection and Flurin Baer, Adrian Baumeyer, Markus Beutle, Rene Buob, Nicole Fischer, Roland Kleger, Stephan Lopez, Gaby Rindlisbacher, Jonas Schaub, Amanda Spillmann, Lukas Staenke, Patrick Wyser, Fabia Wyss, Dominic Wyss, Corinne Zollinger, Reto Lehmann & the technician team for support and assistance at Basel Zoo. We thank Caroline Andrews, Arrate Isasi-Isasmendi and Sebastian Sauppe for providing some of the stimuli. We are also grateful to TTF DataScience NCCR@LiRi for their advice.

## Funding Information

This research was funded by the Swiss National Science Foundation (project grant numbers 310030_185324, KZ, and 100015_182845, BB) and the National Center for Competence in Research “Evolving Language” (SNSF agreement number 51NF40_180888, BB, KZ).

## Author Contributions

SB: Conceptualization; Data curation; Formal analysis; Investigation; Methodology; Project administration; Resources; Visualization; Writing – original draft; Writing – review & editing. PVV: Conceptualization; Methodology; Resources; Writing – review & editing. BB: Conceptualization; Formal analysis; Funding acquisition; Methodology; Resources; Supervision; Visualization; Writing – review & editing. KZ: Conceptualization; Formal analysis; Funding acquisition; Methodology; Resources; Supervision; Visualization; Writing – review & editing.

## DATA AND MATERIALS AVAILABILITY

Data and code are available at https://doi.org/10.60544/wttr-a816.

## Supplementary Material


