# Supplementary material for: Spontaneous Encoding of Event Roles in Hominids
Source: Open Mind (Camb). 2025 Apr 22;9:559–75. doi: 10.1162/opmi_a_00202 (PMC12058332; doi:10.1162/opmi_a_00202)
Supplement: Supplementary file 1 [file opmi-09-559-s001.docx]

**Supplementary Materials for**

***Spontaneous encoding of event roles in hominids***

**This PDF file includes:**

Supplementary Text S1 and S2

Figure S1

Tables S1 to S10

References

**Other Supplementary Materials for this manuscript include the following:**

Dataset S1

Script S1

R Data Files S1 to S9

They are available at: <https://doi.org/10.60544/wttr-a816>

***Supplementary text***

***Text S1: Pilot study***

*Methods*

*Subjects*. Two adult orangutans (1 female (Kila) and 1 male (Bagus); mean 20 ± 1.4 years old; range [19; 21]) participated in the pilot study. Participants were held at Basel Zoo (Switzerland) with their infant male (4 years old). Individuals had access to indoor and outdoor enclosures (814 m^2^). All enclosures contained ropes, hammocks, climbing structures and freshly provided material to build nests. Individuals were fed a mix of fruit and vegetables supplemented with small amounts of proteins, with several feeds distributed throughout the day and free access to water. The study was entirely non-invasive, risk-free and participation was on a fully voluntary basis. The study was approved by the Canton of Basel Veterinary Office (cantonal permit number 3077) and the Animal Welfare Officer at Basel Zoo.

*Stimuli*. A subset of 20 images (from the study set) was used for the test and the 60 same images were used for the training (respectively 5 and 15 images per species depicted).

*Procedure*. Data were collected weekdays from 13:30 to 16:00, from February to June 2022, with the same equipment as the other great apes, with the exception that the setup consisted of a wagon-mounted touchscreen. The touchscreen was also calibrated every day before the start of the session.

*Training phase*. The training procedure was the same as for the study (Fig. 1A-D), with the exception that in the final phase food reward was delivered every five trials.

*Testing phase*. Individual unique lists were generated with similar attention to the order of presentation and with 1,200 trials. Each image was presented 60 times (15 presentations of the same configuration of the stimuli). Each block was divided into five sessions of 60 trials, that were subdivided into sequences of eight trials, after which subjects received food rewards. This subdivision helped to maximise the interest and motivation of the apes. In comparison to Hafri et al. (2018), we opted for fewer stimuli but with more repetition of each, as we feared that apes might lose interest after too many trials.

The testing procedure was quite similar to the study, with the exception that no circle from the targeted colour was presented just before starting the test and that the food reward was delivered every eight trials instead of every two.

*Data processing and analysis.* Trial criteria of exclusion were the same as for the study. On average, 41.05% (SD 3.2%) of trials were excluded per subject (38.79% for Bagus and 43.31% for Kila), primarily resulting from inaccuracies. Similar models to the study were fitted and similar analyses were used, with the exception that the species of the participant was no longer a predictor of interest, instead participants’ identities were used.

*Results*

*Accuracy*. Average accuracy was 75.1% (SD 43.2%) (mean accuracy*_Bagus_* = 77.8% ± 41.6% and mean accuracy*_Kila_* = 72.4% ± 44.7%), after data processing. The best fitting model (Tab. S7 for all comparisons) included as main effect the interaction of the identity of the participant with the repetition of the target side, with robust effect for only one individual (Bagus: marginal effect of repetition of the target side median*_Side Not Repeated – Side Repeated_* = 0.275 odd ratio, 90% CI = [0.191 ; 0.391], meaning that Bagus’ posterior mean odds of correctly choosing the target decreased by approximately 72.5% when the target’s side changed from one trial to the next, posterior median P*_Bagus x Repetition Target Side_* = 0.623, 90% CI = [0.566 ; 0.678], P(*P_Bagus x Repetition Target Side_* > 0.5) = 1; Kila: marginal effect median*_Side Not Repeated – Side Repeated_* = 0.743 odd ratio, 90% CI = [0.511 ; 1.012], posterior median P*_Kila x Repetition Target Side_* = 0.378, 90% CI = [0.324 ; 0.434], P(*P_Kila x Repetition Target Side_* < 0.5) = 1).

*Switch cost effects - spatial continuity and event roles*. The median RT was 1,909.7 ms (RT*_Bagus_* = 2,597.6 ms ± 4,568.4, RT*_Kila_* = 1,403.2 ms ± 1,821.8), after data processing. Model comparison (see Tab. S8 for all model comparisons) demonstrated a robust side switch cost when both individuals were modelled together (β*_Repetition Target Side_*: median = -69.9, 90% CI = [-114.3 ; -25.9], P(β<0) = 1; marginal effect: median*_Side Not Repeated – Side Repeated_* = 140.0, 90% CI = [49.5 ; 226.0]). Participants were on average 485.9 ms (median Δ = 181.0 ms) slower to press on their target when the side switched from one trial to the next. However, this effect was only robust for one individual, when modelled separately (see Tab. S9 for model comparisons; Bagus: marginal effect median*_Side Not Repeated – Side Repeated_* = 245.0, 90% CI = [61.6 ; 431.0]; β*_Repetition Target Side_*: median = -122.5, 90% CI = [-216.0 ; -31.5], P(β<0) = 0.99).

Model comparison also demonstrated that switching from one event role (i.e., agent or patient) to the other from one trial to the next did not influence the RTs, nor did the event roles themselves or the category of the event or the species of the actors (Tab. S8).

*Other effects*. In addition to RTs and the accuracy rates, the side of the response was also of interest as great apes might be prone to side bias. Results showed no side bias (ratio left/right = 1.08 for Bagus and 1.05 for Kila). However, the null model was outperformed by the model including the side of the agent (Δelpd = -2.8, SE*_Δelpd_* = 2.8), with a robust effect (marginal effect: median*_Side Agent Left – Side Agent Right_* = 1.369 odd ratio, 90% CI = [1.137 ; 1.659], meaning that the average posterior odds of pressing on the left side were 1.369 times higher if the agent was on the left side than in the right side; posterior median P*_Side Agent Left_* = 0.578, 90% CI = [0.531 ; 0.622], P(*P_Side Agent Left_* > 0.5) = 1; posterior median P*_Side Agent Right_* = 0.422, 90% CI = [0.377 ; 0.469], P(*P_Side Agent Right_* < 0.5) = 1).

*Discussion*

Overall, the pilot results highlighted that choices and reaction times of at least one individual were influenced by the repetition of the target’s side, but not by the repetition of its role. In addition, both participants’ responses were influenced by the side of the agent on the event displayed on the screen. These findings suggest that participants did process event roles in order to inform their decisions, but they did not demonstrate a role switch cost effect.

Recent studies investigating event role discrimination stressed that social interactions, such as those presented here, triggered more shared attention or preference between the event roles, than did non-social scenes (Brocard et al., 2024; Wilson et al., 2024). These results might explain the absence of a role switch cost in this pilot study, despite participants being sensitive to the agent, as evidenced by their sensitivity to its side, similarly to what was observed for the chimpanzee in the study.

The present pilot study however did not precisely require participants to make a choice, but rather to correctly find a coloured target. In light of this, the accuracy rates of the two orangutans were significantly lower than those observed in Hafri et al. (2018), which recorded an accuracy rate exceeding 95% for human participants. This result could be explained by a lack of attention or a rush to complete a sequence to obtain the food reward. This reasoning is supported by the positive effect that the repetition of the target's side has on reaction time.

Another notable difference from Hafri et al. (2018) is the strikingly longer reaction times of the orangutans participants compared to humans (>3 sec compared to <400 ms, respectively). These slower reaction times might also account for the absence of an event role switching cost. In Hafri et al. (2018), the reported switching cost effects were very small, ranging from 3 to 6 ms and detecting it critically depends on the ability to measure expectations built from one stimulus to the next. However, to access the touchscreen, orangutans had to pass their hand or a stick through the mesh, which slowed them and required their minds to focus on another task, possibly preventing the detection of a switching cost effect or erasing it. However, in light of the study results accuracy of the orangutans was higher than the chimpanzee who was way faster, suggesting a tread of between rapidity and accuracy.

In conclusion, orangutans did not experience a switch cost effect, despite demonstrating some sensitivity to event roles, as evidenced by the influence of the agent’s side on their side choices.

### **Text S2: Target colour difference in humans**

During the pilot, the two individuals tested differed in terms of RTs and accuracy. However, it was unclear if these differences were due to individual’s variations or due to different target’s colour assignment. Thus, the effect of the target’s colour was compared for human participants only.

The colour of the target did not influence the accuracy (mean accuracy blue target = 99.5% ± 7.1%; mean accuracy green target = 99.3% ± 8.6%), as model comparison showed that the colour of the target did not improve the fit of the model to the data in any decisive way (Δelpd*_Null model vs. Model with target colour_* = -0.1, SE*_Δelpd_* = 0.2; marginal effect: median *_Blue – Green_* = 0.906 odd ratio, 90% CI = [0.165 ; 4.665]).

However, the interaction between the target’s colour, the repetition of the target’s side and the repetition of the target’s role influenced the reaction times (Δelpd*_Best model vs. Null model_* = -59.8, SE*_Δelpd_* = 11.5). While participants with the blue target experienced a robust role switch cost (Δ = 6.3 ms; marginal effect: median*_Switched – Repeated_* = 10.9, 90% CI = [5.3 ; 16.4]) participants with the green target did not (Δ = -1 ms; marginal effect: median*_Switched – Repeated_* = 0.8, 90% CI = [-4.9 ; 6.4]). Interaction between role and side continuity also appeared robust in participants with the blue target but not with the green (details in Tab. S10). These differences might be explained by the difficulty, reported by the participants, to detect the green target in a natural greenish environment.

Although the effect of the target’s colour was not robust for all predictors, it seemed to influence some of them. As a result, we chose to take a conservative approach and only included participants assigned the blue target, consistent with the chimpanzee’s assigned colour, for the subsequent analyses.

***Supplementary figure***


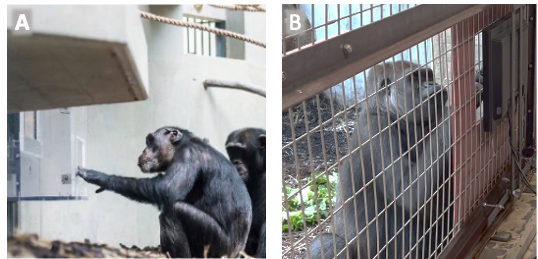


**Figure S1. Different setups used in the study.** (A) fixed setup in the chimpanzees’ enclosure, (B) movable setup in the gorillas’ enclosure. Photo credits: (A) Basel Zoo and (B) S. Brocard.

***Supplementary tables***

**Table S1. Details of the event-category of the stimuli per actor species.**

|  | Chimpanzee | Gorilla | Human | Orangutan |
| --- | --- | --- | --- | --- |
| Grooming | 9 | 5 | 4 | 5 |
| Playing | 1 | 3 | 1 | 2 |
| Embracing | 0 | 1 | 0 | 0 |
| Begging | 0 | 1 | 0 | 0 |
| Pulling | 0 | 0 | 2 | 0 |
| Scratching | 0 | 0 | 1 | 0 |
| Helping | 0 | 0 | 2 | 0 |
| Touching with mouth | 0 | 0 | 0 | 3 |

*Note*. Grooming in humans corresponded to massage, brushing or cutting hairs.

**Table S2. Variables definition.**

| **Variable** | **Name** | **Class** | **Description** |
| --- | --- | --- | --- |
| Accuracy01 | Accuracy of the response | Integer | Accuracy of the response (i.e., selecting the correct target). 2 levels: *0* (incorrect), *1* (correct) |
| Block | Block | Integer | Block in which the stimuli belong to in order of presentation and based on the species of the actors |
| ColAgent | Colour of the agent | Factor | Colour of the agent on the stimuli. 2 levels: *Blue, Green* |
| EventCategory | Category of the event/interaction | Factor | Type of the interaction presented on the image. 8 levels |
| ID | Participant ID name/number | Factor | Name of the great apes or ID number assigned to participant (starting from H01; assigning in sequential order) |
| ID_Species | Participant species | Factor | Species of the participant (*Chimpanzee*, *Human*, and *Orangutan*) |
| ImageName | Name of the stimuli | Factor | Name of the image. 160 levels |
| RepTargetRole | Repetition of the event role of the target | Integer | The event role (i.e., agent or patient) of the target is repeated or switched from one trial to the next. 2 levels: *-1* (role switched), *1* (role repeated) |
| RepTargetSide | Repetition of the side of the target | Integer | The side of the target is repeated or switched from one trial to the next. 2 levels: *-1* (side switched), *1* (side repeated) |
| RoleTarget | Role of the target | Factor | Event role of the target. 2 levels: *Agent, Patient* |
| RT | Reaction times | Numeric | Reaction time of the participant calculated from display of the stimuli to release of the screen |
| SideAgent | Side of the agent | Factor | Side of the agent on the image. 2 levels: *left, right* |
| SideResponse | Side of the response on the screen | Factor | On the image the side of the actor the participant pressed on. 2 levels: *Left, Right* |
| TargetCol | Colour of the target | Factor | Colour of the target along the entire experiment. 2 levels (but one per participant): *Blue, Green* |

**Table S3. Results of the comparison of the expected log pointwise predictive densities (elpd)** of all Bayesian Bernoulli regressions modelling the accuracy of the chimpanzee participant to the study, under the leave-one-out cross validation.

| **Rank model** | **Predictor of interest** | **Difference elpd** | **Difference SE** |
| --- | --- | --- | --- |
| 1 | Role Target | 0.0 | 0.0 |
| 2 | Side Agent | -0.1 | 0.8 |
| 3 | Null | -0.1 | 0.7 |
| 4 | Repetition Target's Role | -0.7 | 1.2 |
| 5 | Repetition Target's Side | -1.4 | 0.7 |
| 6 | Repetition Target's Role + Repetition Target's Side | -1.7 | 1.2 |
| 7 | Repetition Target's Role x Repetition Target's Side | -3.1 | 1.4 |
| 8 | Repetition Target's Role + Repetition Target's Side + Actors' Species + Event Category + Role Target + Agent's Colour + Agent's Side | -3.7 | 1.4 |
| 9 | Repetition Target's Role x Repetition Target's Side + Actors' Species + Event Category + Role Target + Agent's Colour + Agent's Side | -5.4 | 1.5 |

*Note*. The effect of the side of the agent was not robust (median P*_Side Agent = Left_* = 0.537, 90% CI = [0.388 ; 0.684], P(*P_Side Agent = Left_* > 0.5) = 0.65; median P*_Side Agent = Right_* = 0.451, 90% CI = [0.316 ; 0.588], P(*P_Side Agent = Right_* > 0.5) = 0.29; marginal effect median *_Left – Right_* = 1.207 odd ratio, 90% CI = [0.688 ; 2.117]).

**Table S4. Results of the comparison of the expected log pointwise predictive densities (elpd)** of all Bayesian ex-gaussian regressions modelling the reaction times during the study, under the leave-one-out cross validation.

| **Rank model** | **Predictor of interest** | **Difference elpd** | **Difference SE** |
| --- | --- | --- | --- |
| 1 | Repetition Target's Role + Repetition Target's Side + Actors' Species + Event Category + Role Target | 0.0 | 0.0 |
| 2 | Participant's Species + Repetition Target's Role + Repetition Target's Side + Actors' Species + Event Category + Role Target | -0.5 | 0.7 |
| 3 | Participant's Species x Repetition Target's Role + Repetition Target's Side + Actors' Species + Event Category + Role Target | -0.6 | 2.7 |
| 4 | Repetition Target's Role x Repetition Target's Side + Actors' Species + Event Category + Role Target | -0.6 | 0.9 |
| 5 | Participant's Species x Repetition Target's Side + Repetition Target's Role + Actors' Species + Event Category + Role Target | -1.2 | 3.0 |
| 6 | Participant's Species + Repetition Target's Role x Repetition Target's Side + Actors' Species + Event Category + Role Target | -1.3 | 0.9 |
| 7 | Participant's Species x ( Repetition Target's Role + Repetition Target's Side) + Actors' Species + Event Category + Role Target | -2.4 | 3.2 |
| 8 | Participant's Species x Repetition Target's Role x Repetition Target's Side + Actors' Species + Event Category + Role Target | -4.5 | 3.5 |
| 9 | Null | -34.3 | 8.6 |
| 10 | Actors' Species + Event Category + Role Target | -35.4 | 8.6 |

**Table S5. Results of the comparison of the expected log pointwise predictive densities (elpd)** of all Bayesian ex-gaussian regressions modelling the reaction times during the conspecifics’ block of the study, under the leave-one-out cross validation.

| **Rank model** | **Predictor of interest** | **Difference elpd** | **Difference SE** |
| --- | --- | --- | --- |
| 1 | Participant's Species x Repetition Target's Role x Repetition Target's Side | 0.0 | 0.0 |
| 2 | Repetition Target's Side | -4.3 | 5.2 |
| 3 | Participant's Species x Repetition Target's Side | -5.8 | 6.0 |
| 4 | Participant's Species + Repetition Target's Role + Repetition Target's Side | -6.0 | 5.5 |
| 5 | Repetition Target's Role x Repetition Target's Side | -6.5 | 5.4 |
| 6 | Role Target | -10.9 | 6.8 |
| 7 | Null | -10.9 | 6.8 |
| 8 | Participant's Species | -11.0 | 6.8 |
| 9 | Participant's Species x Repetition Target's Role | -11.1 | 6.2 |
| 10 | Repetition Target's Role | -11.3 | 6.9 |

**Table S6. Median marginal effects of the reaction times modeled with the blocks interacting with the target’s side and role switching or repeating.**

|  | **Contrast** | **Estimate** | **90% CI** |
| --- | --- | --- | --- |
| *Target's Role Switched* | Block 1 - Block 2 | 68.1 | [8.7 ; 129.4] |
|  | Block 1 - Block 3 | 107.8 | [17.8 ; 206.2] |
|  | Block 1 - Block 4 | 114.1 | [50.8 ; 175.1] |
|  | Block 2 - Block 3 | 42.4 | [-45.6 ; 121.6] |
|  | Block 2 - Block 4 | 48.9 | [-28.0 ; 115.1] |
|  | Block 3 - Block 4 | 4.8 | [-74.0 ; 91.9] |
| *Target's Role Repeated* | Block 1 - Block 2 | 82.1 | [22.9 ; 143.2] |
|  | Block 1 - Block 3 | 116.3 | [18.5 ; 206.2] |
|  | Block 1 - Block 4 | 143.9 | [81.7 ; 205.3] |
|  | Block 2 - Block 3 | 36.9 | [-47.6 ; 119.2] |
|  | Block 2 - Block 4 | 65.1 | [-10.2 ; 132.2] |
|  | Block 3 - Block 4 | 27.0 | [-51.4 ; 114.2] |
| *Target's Side Switched* | Block 1 - Block 2 | 65.4 | [3.8 ; 124.6] |
|  | Block 1 - Block 3 | 103.4 | [12.5 ; 200.6] |
|  | Block 1 - Block 4 | 130.4 | [67.8 ; 192.0] |
|  | Block 2 - Block 3 | 41.5 | [-42.4 ; 124.8] |
|  | Block 2 - Block 4 | 68.3 | [-6.7 ; 136.3] |
|  | Block 3 - Block 4 | 25.8 | [-57.9 ; 108.2] |
| *Target's Side Repeated* | Block 1 - Block 2 | 85.1 | [21.4 ; 141.5] |
|  | Block 1 - Block 3 | 120.8 | [27.1 ; 215.0] |
|  | Block 1 - Block 4 | 127.8 | [67.4 ; 191.0] |
|  | Block 2 - Block 3 | 38.0 | [-45.9 ; 120.5] |
|  | Block 2 - Block 4 | 45.6 | [-28.9 ; 113.7] |
|  | Block 3 - Block 4 | 6.0 | [-77.4 ; 88.7] |

**Table S7. Results of the comparison of the expected log pointwise predictive densities (elpd)** of all Bayesian Bernoulli regressions modelling the accuracy during the pilot study, under the leave-one-out cross validation.

| **Rank model** | **Predictor of interest** | **Difference elpd** | **Difference SE** |
| --- | --- | --- | --- |
| 1 | ID Participant x Repetition Target's Side | 0.0 | 0.0 |
| 2 | ID Participant x Repetition Target's Side x Repetition Target's Role | -2.3 | 1.9 |
| 3 | ID Participant x Repetition Target's Side x Repetition Target's Role + Actors' Species + Event Category + Role Target | -3.8 | 2.4 |
| 4 | ID Participant + Repetition Target's Side | -4.9 | 3.5 |
| 5 | ID Participant + Repetition Target's Side x Repetition Target's Role | -5.4 | 3.9 |
| 6 | ID Participant + Repetition Target's Side + Repetition Target's Role | -5.5 | 3.7 |
| 7 | ID Participant + Repetition Target's Side + Repetition Target's Role + Actors' Species + Event Category + Role Target | -6.9 | 4.0 |
| 8 | Repetition Target's Side | -8.4 | 4.3 |
| 9 | ID Participant | -19.8 | 6.3 |
| 10 | ID Participant + Repetition Target's Role | -20.5 | 6.4 |
| 11 | ID Participant x Repetition Target's Role | -21.9 | 6.4 |
| 12 | Null | -22.9 | 6.6 |
| 13 | Repetition Target's Role | -23.8 | 6.7 |

**Table S8. Results of the comparison of the expected log pointwise predictive densities (elpd)** of all Bayesian ex-gaussian regressions modelling the reaction times during the pilot study, under the leave-one-out cross validation.

| **Rank model** | **Predictor of interest** | **Difference elpd** | **Difference SE** |
| --- | --- | --- | --- |
| 1 | Repetition Target's Side | 0.0 | 0.0 |
| 2 | Repetition Target's Side + Actors' Species + Event Category + Role Target | -3.8 | 1.9 |
| 3 | ID Participant + Repetition Target's Side + Actors' Species + Event Category + Role Target | -4.6 | 2.1 |
| 4 | Repetition Target's Role + Repetition Target's Side + Actors' Species + Event Category + Role Target | -4.8 | 1.9 |
| 5 | Actors' Species + Event Category + Role Target | -5.8 | 3.5 |
| 6 | ID Participant + Repetition Target's Role + Repetition Target's Side + Actors' Species + Event Category + Role Target | -5.9 | 2.1 |
| 7 | ID Participant + Actors' Species + Event Category + Role Target | -6.3 | 3.7 |
| 8 | ID Participant + Repetition Target's Role x Repetition Target's Side + Actors' Species + Event Category + Role Target | -6.4 | 2.3 |
| 9 | ID Participant x (Repetition Target's Role + Repetition Target's Side) + Actors' Species + Event Category + Role Target | -6.5 | 2.8 |
| 10 | Repetition Target's Role + Actors' Species + Event Category + Role Target | -6.8 | 3.5 |
| 11 | ID Participant + Repetition Target's Role + Actors' Species + Event Category + Role Target | -7.2 | 3.7 |
| 12 | ID Participant x Repetition Target's Role x Repetition Target's Side + Actors' Species + Event Category + Role Target | -7.6 | 3.0 |

**Table S9. Results of the comparison of the expected log pointwise predictive densities (elpd)** of all Bayesian ex-gaussian regressions modelling the reaction times of both individuals modelled separately during the pilot study, under the leave-one-out cross validation.

| **ID** | **Rank model** | **Predictor of interest** | **Difference elpd** | **Difference SE** |
| --- | --- | --- | --- | --- |
| Bagus | 1 | Repetition Target's Side + Actors' Species + Event Category + Role Target | 0.0 | 0.0 |
|  | 2 | Repetition Target's Role + Repetition Target's Side + Actors' Species + Event Category + Role Target | -0.3 | 1.1 |
|  | 3 | Repetition Target's Role x Repetition Target's Side + Actors' Species + Event Category + Role Target | -0.6 | 1.2 |
|  | 4 | Actors' Species + Event Category + Role Target | -0.9 | 2.2 |
|  | 5 | Repetition Target's Role + Actors' Species + Event Category + Role Target | -2.1 | 2.3 |
| Kila | 1 | Actors' Species + Event Category + Role Target | 0.0 | 0.0 |
|  | 2 | Repetition Target's Side + Actors' Species + Event Category + Role Target | -0.2 | 1.5 |
|  | 3 | Repetition Target's Role + Repetition Target's Side + Actors' Species + Event Category + Role Target | -0.3 | 1.8 |
|  | 4 | Repetition Target's Role + Actors' Species + Event Category + Role Target | -0.5 | 1.0 |
|  | 5 | Repetition Target's Role x Repetition Target's Side + Actors' Species + Event Category + Role Target | -1.9 | 2.0 |

*Note.* Bagus had the blue target and was a male, while Kila had the green target and was a female.

**Table S10. Median RTs across human participants** according to the colour of their target and separately for all factors of the best fitting model.

| Target colour | Condition | Reaction times (ms ± SD) | | Switch cost (ms) | Median marginal effect (90% CI) |
| --- | --- | --- | --- | --- | --- |
|  |  | Repeated | Switched |  |  |
| Blue | Role | 744.3 (348.5) | 750.6 (346.8) | 6.3 | 10.5 [4.8 ; 16.0] |
|  | Side | 723.3 (342.4) | 777.4 (352.1) | 54.1 | 30.7 [24.8 ; 36.1] |
|  | Role, Side repeated | 721.3 (345.3) | 723.8 (339.5) | 2.5 | 9.6 [1.6 17.3] |
|  | Role, Side switched | 773.5 (351.9) | 781.8 (352.9) | 8.3 | 12.2 [4.3 ; 20.2] |
| Green | Role | 756.1 (332.3) | 755.1 (323.9) | -1.0 | 0.1 [-5.5 ; 5.7] |
|  | Side | 734.3 (322.1) | 778.8 (333.2) | 44.4 | 21.9 [16.1 ; 27.5] |
|  | Role, Side repeated | 736.0 (328.3) | 730.0 (315.8) | -6.0 | -5.5 [-13.1 ; 2.6] |
|  | Role, Side switched | 779.6 (335.6) | 777.1 (330.8) | -2.5 | 7.2 [-1.0 ; 15.1] |

*Note*. Median marginal effects are extracted from the best model (Target colour x Repetition Target's Role x Repetition Target's Side) and are the difference between Switched and Repeated. Marginal effects for simple interaction were extracted for models without the triple interaction to avoid misleading results. The switch cost is the difference between RT switched and repeated.

***References***

Brocard, S., Wilson, V. A. D., Berton, C., Zuberbühler, K., & Bickel, B. (2024). A universal preference for animate agents in hominids. *iScience*, *27*(6), 109996. https://doi.org/10.1016/j.isci.2024.109996

Hafri, A., Trueswell, J. C., & Strickland, B. (2018). Encoding of event roles from visual scenes is rapid, spontaneous, and interacts with higher-level visual processing. *Cognition*, *175*, 36–52. https://doi.org/10.1016/j.cognition.2018.02.011

Wilson, V. A. D., Sauppe, S., Brocard, S., Ringen, E., Daum, M. M., Wermelinger, S., Gu, N., Andrews, C., Isasi-Isasmendi, A., Bickel, B., & Zuberbühler, K. (2024). Humans and great apes visually track event roles in similar ways. *PLOS Biology*, *22*(11), e3002857. https://doi.org/10.1371/journal.pbio.3002857
